# Characterising the Multiple‐Plastic Degrading Strain of *Bacillus subtilis* GM_03 From the *Galleria mellonella* Microbiome

**DOI:** 10.1111/1758-2229.70216

**Published:** 2025-10-20

**Authors:** Fiona S. B. Facey, Ram Maharjan, Hue Dinh, Jason S. Buchanan, Luke A. Connal, Aidan P. Tay, Ian T. Paulsen, Amy K. Cain

**Affiliations:** ^1^ School of Natural Sciences Macquarie University North Ryde New South Wales Australia; ^2^ ARC Centre of Excellence in Synthetic Biology, School of Natural Sciences Macquarie University North Ryde New South Wales Australia; ^3^ Research School of Chemistry Australian National University Canberra, ACT Australia

**Keywords:** *Bacillus subtilis*, biodegradation, *Galleria mellonella*, polyethylene, polyurethane

## Abstract

Plastic waste is a mounting global problem with over 400 million tons of plastic produced annually and over 50% ending up in landfill after its intended use. Two types of plastics are particularly problematic and are difficult to recycle: low‐density polyethylene (LDPE) and polyurethane (PU). Fortuitously, nature may offer a potential solution; *Galleria mellonella* larvae can digest various plastics, including LDPE, which is believed to be driven by microbes in their gut microbiome. Although some studies have examined their gut microbiota on a metagenomic level, little is known about their ability to degrade plastics. Here, we isolated six bacterial strains from *G. mellonella* larvae feeding on LDPE. One of them, identified as *Bacillus subtilis* GM_03, has the capacity to break down commercial PU (Impranil), in addition to LDPE. This bacterium encodes a suite of genes required for plastic degradation. Directed evolution was used to enhance this strain's plastic degrading rate by over six‐fold. Sequencing of the evolved culture revealed four genes, *srfAB*, *fadD*, *appA* and *citS*, associated with this increased PU degradation rate. This is the first time that *B. subtilis* isolated from *G. mellonella* larvae has been shown to be capable of degrading multiple types of plastics.

## Graphical abstract

A novel strain of *Bacillus subtilis* has been isolated from plastic‐fed *Galleria mellonella* larvae, which shows evidence of degradation of both LDPE and PU plastics. Genes potentially associated with plastic degradation have been identified following directed evolution and genome sequencing of the strain.

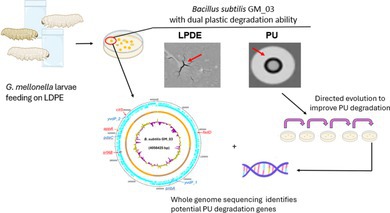

## 1 Introduction

Plastic waste is a major global problem, with over 400 million tonnes of plastic being produced annually as of 2022 (OECD 2022) and less than 10% of this being recycled (OECD 2022; Plastics Europe 2022). It has been predicted that without change, there will be approximately 12 million tonnes of plastic waste generated per annum (Geyer et al. 2017), in part due to the lifespan of more than half of plastics being less than 1 month (Nayanathara Thathsarani Pilapitiya and Ratnayake 2024). Seventy percent of the approximately 370 M tonnes of fossil‐based plastic manufactured in 2023 is comprised of only five plastic types (polypropylene, polyethylene [PE], polyvinyl chloride, polyethylene terephthalate and polyurethane [PU]) (Plastic Europe 2025). The common usage of plastics is due to properties such as high strength, versatile functionality and low cost to manufacture (Nayanathara Thathsarani Pilapitiya and Ratnayake 2024), with around 40% of global plastics being used as packaging materials (Damayanti et al. 2022). Plastic pollution is clearly a major environmental issue, with estimates of close to 10 million tonnes of plastic entering the oceans annually (Lau et al. 2020). Plastic pollution in aquatic and terrestrial environments destabilises ecosystems, disrupts natural carbon and nutrient cycles and microplastics can enter the food chain, ultimately harming human and animal health (Haney and Rochman 2025; MacLeod et al. 2021).

PE is the most widely used synthetic plastic, accounting for approximately 27% of all plastics produced (Plastics Europe 2022). PE is commonly used in household items such as plastic bags and food wrap and containers (Ghatge et al. 2020). It is a thermoplastic that does not change its chemical composition upon heating (Amobonye et al. 2021) and the carbon backbone, which does not readily undergo hydrolysis, renders it recalcitrant to degradation (Chamas et al. 2020). The qualities that make it attractive for use in packaging, namely its strength and chemical resistance, subsequently enable its perseverance in the environment (Menzel et al. 2022). The low‐density form of PE (LDPE) accounts for over half of the PE manufactured today and is widely considered the most difficult plastic to recycle.

PU is a major form of industrially used plastic, with a market size of $74B USD in 2023. (Gama et al. 2024), almost one third of the waste that is generated is disposed of in landfill sites (Gama et al. 2024). PU is a polymer with a combination of unique features (namely durability, lightweight, flexibility and high strength) that make it incredibly valuable across diverse areas including manufacturing, medicine, fashion and automotive industries. PU is a thermoset plastic, which, in contrast to the thermoplastic PE, has irreversible changes upon heating, meaning that PU plastics tend to lose integrity and are downgraded after incineration (Amobonye et al. 2021). Many forms of PU are built to be long‐lasting plastics, like those found in mattresses, cushions and paints, and therefore are designed to be difficult to break down. Finally, many plastic waste streams are comprised of mixed plastics, and very few recycling solutions can target mixed waste, but natural solutions such as microbes might hold promise in this area.

In this study, we explored avenues to advance recycling of the difficult‐to‐recycle plastics LDPE and PU by exploiting the natural talents of plastic eating microbes. Multiple bacteria have previously been identified that can degrade LDPE, including *Bacillus licheniformis* (Saeed et al. 2022). *Pseudomonas* sp. (Tribedi and Sil 2013) and *Bacillus subtilis* (Sudhakar et al. 2008). There have also been reports of bacterial degradation of PU by species including *Pseudomonas protegens* (Biffinger et al. 2014), *Acinetobacter gerneri* P7 (Howard et al. 2012) and *Pseudomonas putida* (Peng et al. 2014). Also, the larvae of the greater wax moth *Galleria mellonella* are honeybee pathogens that invade hives and have evolved to consume the beeswax inside (Kwadha et al. 2017), which has structural similarities to long‐chain alkanes found in plastics (Kong et al. 2019). Many studies have shown that *G. mellonella* larvae are uniquely able to efficiently break down multiple synthetic plastics, including LDPE and PU (Bombelli et al. 2017; LeMoine et al. 2020; Barrionuevo et al. 2022; Boschi et al. 2023). There are various hypotheses regarding the specific mechanisms by which *G. mellonella* veraciously consume plastics and most studies have focussed on one single plastic (LDPE) (Sanluis‐Verdes et al. 2022; Cassone et al. 2022; Poma et al. 2022). Many studies have focussed on examining enzymes produced by the caterpillar host itself (Kong et al. 2019; Sanluis‐Verdes et al. 2022). Other studies have taken a metagenomics‐based approach to observe overall shifts in gut microbiota during plastic consumption (Cassone et al. 2020; Lou et al. 2020). However, the few that have taken a functional approach to characterising isolated microbes from the gut microbiome, after host plastic consumption, found *Acinetobacter* , *Enterobacter* and *Bacillus* spp. (LeMoine et al. 2020; Yang et al. 2014) implicated with plastic‐degradation.

Our hypothesis and the focus of this study is that key microbes contained within the gut microbiome of the insect are important drivers of plastic degradation, and that these may have broader, cross‐plastic degrading capabilities like their larval hosts. Investigating the mechanisms behind the plastic‐degrading potential of *G. mellonella* could provide critical insights for developing innovative biodegradation strategies and reducing the environmental burden of plastic waste. This may allow us to harness evolved naturally occurring strategies to tackle breaking down and recycling stubborn plastics, like LDPE and PU. In this study, we have isolated bacterial species from plastic‐fed *G. mellonella* larvae and assessed their ability to degrade LDPE and PU. A strain of *B. subtilis* (GM_03) was shown to be able to break down both LDPE and PU and hence was further characterised using genomics, phylogenetics and directed evolution.

## 2 Materials and Methods

### 2.1 Isolating PE‐Degrading Bacteria From Great Wax Moth Larvae

*G. mellonella* larvae were reared in a controlled environment at the Galleria Research Facility, Macquarie University, Australia at 30°C and 50% humidity with a 12 h light, 0.5 h dusk and 11.5 h dark cycle, as previously described (Frei et al. 2021). Larvae from the egg stage were fed with a 2:1 mixture of standard diet (rice cereal [50.2%], glycerol [23.3%], honey [23.3%] and baker's yeast [3.2%]), as previously described (Firacative et al. 2020) and yellow beeswax obtained from the apiaries at the School of Natural Sciences, Macquarie University to enrich the gut microbiome with long‐chain hydrocarbon metabolising bacterial species. Larvae of the required size ~10 to 20 mm in length were removed from the maintenance colony, and groups of five larvae were placed into sterile Petri dishes. After starving the groups for 24 h, three groups of larvae were only fed sterile 23 μm LDPE (Caspak) in 10 × 10 mm squares. Larvae were incubated for a further 4–5 days and harvested before pupation to isolate potential PE‐degrading bacteria, and following anesthetising at −30°C for 5 min, the external surface of each larva was sterilised by submersion in 80% v/v ethanol for 1 min, rinsed twice with sterilised 1 M phosphate buffered saline (PBS), followed by homogenisation in 1.5 mL microfuge tubes containing 200 μL of sterile PBS using a micro pestle.

Larval homogenates were serially diluted using PBS at 10^−1^ and 10^−2^ dilutions and inoculated onto sterile M9‐LDPE minimal salts agar plates supplemented with 0.5% w/v LDPE. Briefly, LDPE‐M9 agar plates were prepared as follows: A total of 2 g of LDPE powder (500 μm size, Alfa Aesar) was solubilised in 32 mL of paraffin oil at ~80°C for 20 min, followed by the addition of 80 mL of 0.05% v/v Tween 20 to assist dispersion of LDPE. After the solution appeared homogeneous, 200 mL of 1.5% w/v sterilised agar was added to the solution, followed by 80 mL of 5 × M9 minimal salts media (KH_s_P0_4_, 3 g/L, NaCl 0.5 g/L, Na_2_HPO_4_ 6.78 g/L, NH_4_Cl 1 g/L), pH 7.0, supplemented with 0.1 mM CaCl_2_ and 2 mM MgSO_4_. M9‐LDPE minimal salt agar plates without inoculum and M9 minimal salt agar served as negative controls. All plates were incubated at 30°C and 37°C to enrich for mesophilic bacteria capable of survival on LDPE for 7 days.

### 2.2 Confirmation of LDPE Degradation by SEM

A total volume of 25 mL of M9 minimal salts media supplemented with 0.1 mM CaCl_2_ and 2 mM MgSO_4_ was inoculated with approximately 1 × 10^8^ cells and a sterile 50 × 50 mm square of LDPE sheet as the sole carbon source and incubated at 37°C, 200 rpm for 95 days, followed by removal of bacteria with 2% SDS for 4 h. Pieces of 8 × 8 mm were cut for scanning electron microscopy (SEM), mounted on standard aluminium SEM pin stubs, 8 mm diameter with double‐sided carbon tape. Samples were coated in gold at ~20 nm thickness using the Emitech K550 Gold Sputter Coater. LDPE surfaces before and after incubation with bacterial cultures were examined using PHENOM XL Benchtop SEM under high vacuum at 10 kV.

### 2.3 Strain Identification Using 16S rRNA Sequencing

Five to ten colonies from individual bacteria were inoculated into 50 μL of sterile double distilled water and heated at 97°C for 5 min, followed by centrifugation at 5600 × *g* for 2 min. The supernatant was retained as template DNA for PCR. 16S rRNA PCR was performed using universal 16S rRNA primers 27F 5′‐AGAGTTTGATCCTGGCTCAG‐3′ and 1492R 5′‐ACGGCTACCTTGTTACGACTT‐3′ (IDT) as previously published (Heuer et al. 1997). Briefly, each PCR was carried out in 50 μL reactions containing 25 μL of 2× PCR Master Mix (Thermo Scientific), 21 μL of Nuclease Free Water (Thermo Scientific), 1 μL of forward and reverse primers, and 1 μL template DNA, followed by Sanger Sequencing (Macrogen, Korea). Sequences obtained were checked using quality control (QC) and trimmed to remove reduced quality reads in MEGA11 (Tamura et al. 2021). Nucleotide sequences were identified using the Basic Local Alignment Search Tool (Altschul et al. 1990) for nucleotides (BLASTn) and deposited in the National Center for Biotechnology Information (NCBI) nucleotide database under accession numbers: PV224562, PV224564, PV224566, PV224569, PV224570, PV224571.

### 2.4 Impranil Clearance Assay

For PU degradation assay, we used M9 minimal medium agar plates supplemented with 3 g/L Impranil (ImpranilDLN), and 20 mM sodium citrate was used as described previously (Hung et al. 2016). The six isolates obtained from *G. mellonella* were revived from glycerol stocks on Mueller Hinton (MH) (pH 7.3) agar plates and resuspended in sterile PBS to give an OD_600nm_ of 1 before pipetting 20 μL spots and incubating at 37°C. Plates were then monitored for up to 13 days for clearance zones indicating degradation of Impranil.

### 2.5 Survival Assays of *B. subtilis* GM_03 in Liquid Cultures Containing PU and LDPE

For the survival assay, a total volume of 10 mL of M9 minimal salts media containing 1% LDPE powder with a particle size of 500 μm as the sole carbon source was inoculated with 1 × 10^8^ overnight grown *B. subtilis* GM_03 and incubated at 37°C with 200 rpm for 166 days. Samples were taken to monitor the bacteria survival at time intervals of 6 to 24 days. Culture samples were serially diluted and plated on MH plates and incubated at 37°C. Cell viability was then estimated by counting surviving colonies. Seven technical replicates were taken for each sample to count the number of colonies forming units (CFU). For monitoring survival of *B. subtilis* GM_03 in PU, M9 medium containing Impranil at a final concentration of 3 g/L was used, and survival of cells was monitored as in LDPE.

### 2.6 Assessment of LDPE Degradation by Tensile Testing

A total volume of 25 mL of M9 minimal salts media supplemented with 0.1 mM CaCl_2_ and 2 mM MgSO_4_ was inoculated with approximately 1 × 10^8^ cells and a sterile 50 × 50 mm square of LDPE sheet as the sole carbon source and incubated at 37°C, 200 rpm for 95 days, followed by the removal of bacteria with 2% SDS for 4 h. The film samples were prepared for tensile testing at the Research School of Chemistry at Australian National University, cut into samples of length, width, and thickness 7.41 ± 0.29 mm, 5.89 ± 0.52 mm and 0.23 ± 0.01 mm respectively. Width and thickness were measured before the samples were fitted to the test stand and force gauge. The samples were held taut at which point the length of the film was measured as the distance between the top and bottom clamps. The stand was raised at a rate of 10 mm/min while changes in force were recorded, with recording stopped upon the breakage of the sample.

Ultimate tensile strength (*σT*) was calculated as the peak force (FP) divided by the area (*A*), where area is the width multiplied by the thickness of the sample.

σT=FP/A

Toughness was obtained using the Integrate function in OriginPro 2023b to calculate the area under the stress–strain curve of each sample. The error was calculated as the standard deviation for the five replicates. Statistical analysis was carried out using a paired student's *t*‐test, with a confidence level of 95% in GraphPad Prism version 10.0.0 for Windows, GraphPad Software, Boston, Massachusetts, USA.

### 2.7 Directed Evolution Experiments of *B. subtilis* GM_03

To evaluate the potential for enhancing PU degradation of *B. subtilis* GM_03 by directed evolution, we employed a serial passaging approach based on previously published methodology (Cain et al. 2018). Parental *B. subtilis* GM_03 was grown overnight in 5 mL of M9 citrate media at 37°C with shaking at 200 rpm. Twenty microlitres of cultures containing 10^6^ cells were spotted on M9 citrate‐agar plates supplemented with 3 g/L Impranil and incubated at 37°C. The time taken for the formation of a clearance zone of approximately 0.5 mm indicating PU degradation was monitored. Once a clearance zone was formed, bacterial populations from the spot were collected, resuspended in sterile PBS, and a 20 μL culture containing 10^6^ cells was spotted onto a fresh M9 citrate‐Impranil plate. The time required to form an initial clearance zone (measuring at least 0.5 mm) was monitored. This process was repeated for four successive passages, reducing the clearance zone formation time from 13 days to 2 days by the fourth passage.

### 2.8 Whole‐Genome Sequencing and Bioinformatic Analysis

DNA was isolated from cultures of the *B. subtilis* GM_03 parental strain and the passage four of the directed evolution experiment, grown overnight at 37°C with 200 rpm shaking, according to the protocol of the DNeasy UltraClean Microbial Kit (Qiagen). Whole‐genome sequencing on the Illumina Novaseq 6000 using the Illumina DNA prep Library was performed by the Australian Institute for Microbiology & Infection (AIMI), University of Technology, Sydney. The genome was assembled using Shovill 1.0.9 (https://github.com/tseemann/shovill) and visualised using Bandage (Wick et al. 2015) and was annotated using Prokka 1.14.6 (Seemann 2014), visualising in the Artemis genome browser (Carver et al. 2011). For identification of plastic degrading enzymes, all available LDPE‐, PU‐, and Impranil‐degrading protein sequences were downloaded from the Plastic Biodegradation Database (PlasticDB) (Gambarini et al. 2022) and any sequence homology between these and *B. subtilis* GM_03 was assessed using BLASTp (Altschul et al. 1990).

Mutations were identified between the parental isolate and evolved passage 4 population using SNIPPY (SNIPPY 2015) and mutations identified were further confirmed using BLASTn (Altschul et al. 1990). The complete genomes of 342 strains of *B. subtilis* were downloaded, and FastANI (Jain et al. 2018) was used to calculate the average nucleotide identity. The output from FastANI was used to construct a distance matrix and phylogenetic tree, as previously described (Semenec et al. 2023).

## 3 Results

### 3.1 Isolation of Bacterial Isolates and Preliminary Screening for LDPE and PU Degradation

**FIGURE 1:**
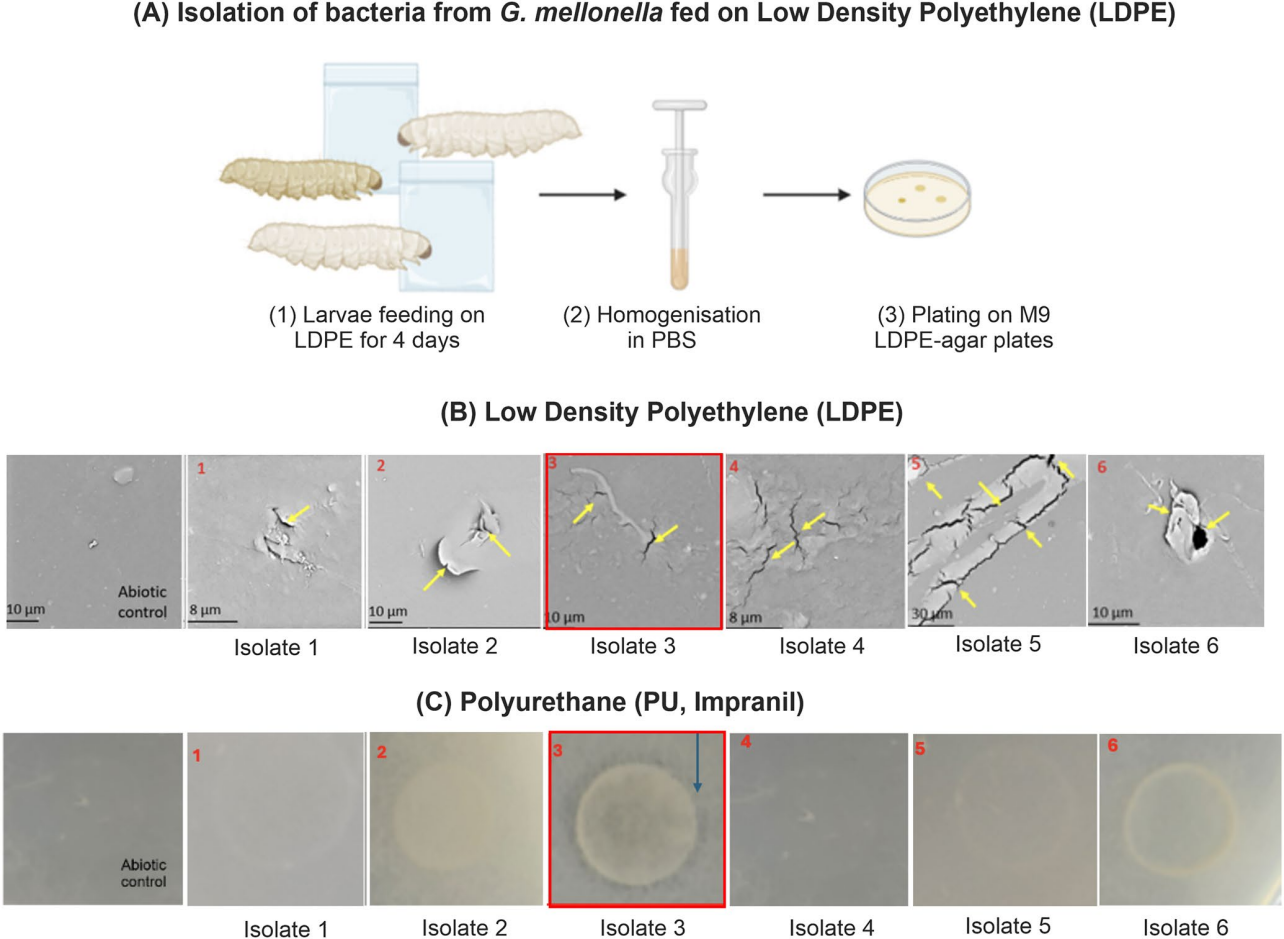
(A) Methodology of obtaining bacterial isolates from *G. mellonella larvae*: Following feeding with LDPE, larvae were sterilised in 80% ethanol and homogenised in sterile PBS with homogenate inoculated onto agar plates containing 500 µm LDPE powder and incubated. Colonies showing growth were isolated. (B) SEM images of LDPE film after incubation in M9 minimal media with 50 × 50 mm LDPE film with 6 isolates collected from *G. mellonella* larvae. Incubation took place for 95 days at 37°C and 200 rpm. Yellow arrows indicate areas of cracking and pitting on the PE. Representative images are shown only with six images captured for each sample. (C) Identification of an Impranil‐degrading bacterial strain from *G. mellonella* . Arrow indicates the clearance zone formed by bacterial Isolate 3. Identification from 16S rRNA sequencing: 1. *Dermacoccus* sp., 2. *Bacillus* sp., 3. *Bacillus* sp., 4. *Dermacoccus* sp., 5. *Enterococcus* sp., 6. *Micrococcus* sp.

We obtained six microbial isolates from cultures derived from *G. mellonella* larvae that had been fed a LDPE‐only diet for 4 days (Figure 1A). We identified these microbes to a genus level using 16S rRNA sequencing and BLASTn alignment, including *Dermacoccus* , *Enterococcus* , *Micrococcus* and *Bacillus* spp. The presence of these diverse bacterial genera suggests that the *G. mellonella* digestive tract is colonised by a wide range of bacterial species, in line with other studies investigating the microbial composition of the *G. mellonella* gut (Cassone et al. 2020; Gohl et al. 2022). The 16S rRNA sequences of these bacteria have been deposited in the NCBI GenBank nucleotide database under the following accession numbers: PV224562, PV224564, PV224566, PV224569, PV224570 and PV224571 (Table S1).

The six isolates were screened for their capacity to degrade LDPE as well as the additional plastic PU. For LDPE biodegradation, we cultured bacteria in M9 minimum medium with submerged LPDE squares (5 cm × 5 cm) for 95 days. PU biodegradation was evaluated by using the M9 citrate agar plates supplemented with 3 g/L Impranil method, as previously described (Hung et al. 2016; Rowe and Howard 2002; Biffinger et al. 2015). After 95 days of incubation of LDPE squares together with bacteria, SEM imaging was performed to check for evidence of LDPE degradation. We observed areas of cracks and pitting in all treated samples while no such change was found in the untreated samples incubated in M9 salts alone (Figure 1B). We next assessed the PU clearance ability of the six LDPE‐degrading microbes, compared to a non‐treated abiotic control (Figure 1C). Among the 6 isolates tested, only ‘Isolate 3’ was able to degrade PU, as evidenced by the formation of a PU clearance zone around the bacterial inoculation spot, and we focused on characterising this isolate further (marked red in Figure 1C).

### 3.2 Characterisation of *B. subtilis* GM_03 With Dual‐Plastic Degrading Ability

**FIGURE 2:**
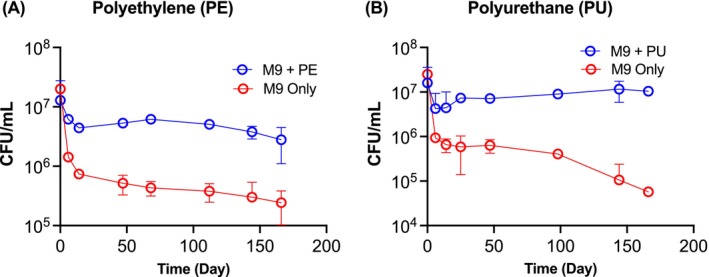
Cell viability of *B. subtilis* GM_03 in the presence of low‐density polyethylene (LDPE) and Impranil (PU). Cell viability assays were performed by estimating colony‐forming units (CFU) in M9 medium in the presence of 500 μm LDPE powder (A) and polyurethane Impranil PU (B) as the only carbon source over 166 days.

Isolate 3, which uniquely demonstrated the ability to degrade both LDPE and PU, was further assessed for its capacity to utilise these plastics as a growth substrate using plastic survival assays. Survival curve results showed that although the number of cells dropped initially due to the stressful conditions, ‘Isolate 3’ survived significantly longer in cultures containing LDPE or PU than in M9 controls (Figure 2). Further, the ability of ‘Isolate 3’ to degrade LDPE film was analysed using a uniaxial tensile tester, which measures ultimate tensile strength and toughness of the LDPE. We observed a general decrease in both the toughness and ultimate tensile strength when Isolate 3 was inoculated on LDPE sheets, compared to the untreated control (Figure S1). Although the error range for the treated sample is quite large at *ca*. 46% and 37% respectively, this variation may be expected as the physical properties of the treated LDPE samples would vary compared to the pristine controls. We speculate this may be caused by non‐uniform attack by the bacteria, such as cracking and pitting (Figure 1B), leading to inhomogeneous degradation.

**FIGURE 3:**
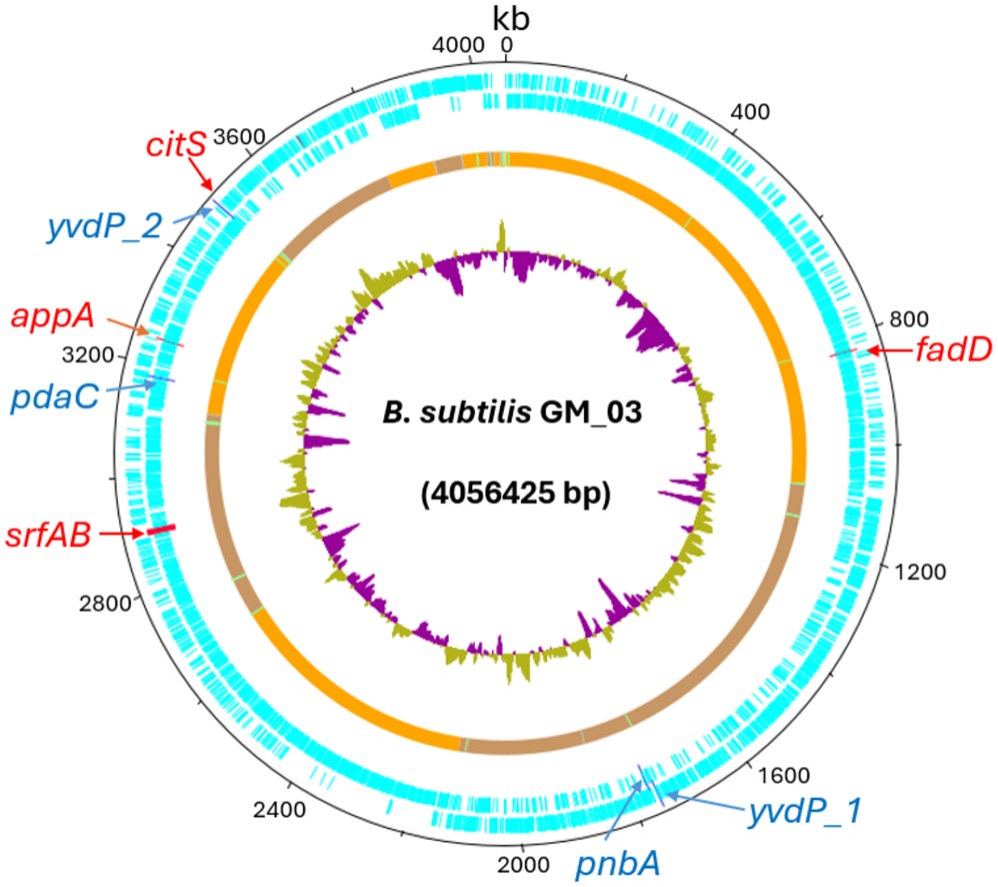
DNA plot of *B. subtilis* GM‐03 genome, highlighting genes that potentially associated with PU and LDPE degradation activity. Genes present in *B. subtilis* GM‐03 that also overlap with PlasticDB are in dark blue types. Genes identified through metagenomic sequencing with mutations potentially associated with enhanced plastic degradation are highlighted in red types. The GC content is depicted in green (indicating regions of low GC content) and purple (indicating regions of high GC content). Numbers on the outer track represent genome positions.

Given its unique capability to degrade multiple plastics, we genotypically characterised this isolate by conducting whole‐genome sequencing. Through this, we confirmed its identity as *B. subtilis* and designated the strain as *B. subtilis* GM_03 (Sequence deposited in the European Nucleotide Archive (ENA) under the accession number: ERR14648077). This finding aligns with previous reports observing that the *Bacillus* genus is highly active in the *G. mellonella* microbiome when larvae were fed with plastics (Cassone et al. 2022; Gohl et al. 2022). The genome of *B. subtilis* GM_03 contained 4,056,425 base pairs with 44.56% GC content and was assembled in 24 contigs. Next, we annotated the genome with PROKKA (Seemann 2014), revealing 4022 predicted coding sequences, as visualised in Artemis DNA plotter (Figure 3).

We next identified any potential plastic degrading genes (shown in blue, Figure 2) by aligning all proteins of *B. subtilis* GM_03 against the PlasticDB (Gambarini et al. 2022) using BLASTp (Altschul et al. 1990). We filtered the top matches by taking an amino acid identity of > 30% and an *E*‐value cut‐off of < 10^−5^, and four genes were identified as having high similarity and likely plastic degrading activity. Specifically, there were three enzymes shown to catalyse PU degradation (including two oxidoreductases and an esterase) and one enzyme for LDPE degradation (a deacetylase) (Figure 3 and Table S2). These findings highlight the potential environmental adaptability and functional diversity of *B. subtilis* GM_03.

To assess the relatedness of the GM_03 strain isolated from *G. mellonella* to broader, global *B. subtilis* isolates, we aligned this genome with the 342 *B. subtilis* genomes available from the NCBI non‐redundant nucleotide database, analysed using an ANI similarity matrix and visualised as a phylogenetic tree (Figure S2). This phylogenetic analysis revealed the two most closely related strains to GM_03 originated from soil but were geographically divergent, being isolated in the United States and Belarus. *B. subtilis* JM553 (accession: CP160220) was isolated from the rhizosphere of the cotton plant and, importantly, *B. subtilis* BIM B‐569G (accession: NZ_CP069789) was used as a model organism to study the biodegradation of the hydrocarbon crude oil (Klishevich et al. 2018).

### 3.3 Directed Evolution of *B. subtilis* to Enhance Degradation of PU

**FIGURE 4:**
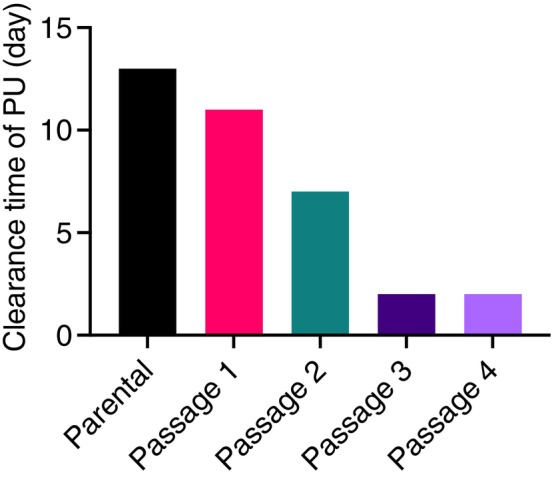
Clearance time of PU Impranil by *B. subtilis* GM_03. Bar graph indicates the number of days to initial clearance from parental strain through to Passage 4 with reduction in time for initial clearance zone (0.5 mm) from 13 to 2 days.

The unique capability of *B. subtilis* GM_03 to degrade both LDPE and PU means that it could be a promising candidate for biotechnological applications in plastic recycling. Therefore, we sought to determine whether we could improve the rate of its plastic degradation. To enhance its PU degradation capacity, we applied a directed evolution approach by serial passaging in M9 citrate‐agar media supplemented with 3 g/L Impranil over 5 weeks (see experimental diagram in Figure S3). The time taken to show an initial clearance zone of 0.5 mm in the Impranil‐containing agar plates was reduced by 6‐fold, from 13 days in the parental strain to only 2 days in the Passage 4 strain (Figure 4). This finding suggests that *B. subtilis* GM_03 rapidly adapts to PU‐containing environments and could have potential for optimised use in industrial applications.

To identify genetic mutations responsible for adaptation in PU containing media, we conducted population‐level comparative genome sequencing of the Passage 4 culture (Sequence deposited in ENA with accession number: ERR14648078), compared to the parental (un‐passaged) strain. This analysis identified mutations in four genes: *fadD* (long chain fatty acid CoA ligase), *appA* (oligopeptide binding protein), *citS* (two‐component sensory histidine kinase) and *srfAB* (surfactin synthetase subunit 2) (Table 1). Interestingly, we found a total of 11 different mutations in *srfAB* genes (Table 1), suggesting this gene was heavily selected for in the PU containing conditions.

**TABLE 1:** List of mutations detected in the Passage 4 citrate‐Impranil‐evolved population of *Bacillus subtilis* GM_03 relative to the parental strain.

Gene	Product or function	Mutation	Codon change	Type^a^	Position in gene^b^	Frequency	Amino acid change and position in protein
*fadD*	Long chain fatty acid CoA ligase	G to A	GGT to GAT	nsSNP	1229	100%	G410D
*citS*	Two component sensory kinase	G to A	GAG to AAG	nsSNP	937	100%	E313K
*appA*	Oligopeptide binding protein	A to AA	Frameshift	Insertion	496	93%	—
*sfrAB*	Surfactin synthase subunit 2	A to G	GTA to GTG	sSNP	8871	89%	na
		T to C	AAT to AAC	sSNP	8898	84%	na
		C to A	AAT to AAC	sSNP	8922	84%	na
		A to T	ACA to TCA	nsSNP	8929	83%	T2977S
		T to C	GAT to GAC	sSNP	8937	83%	na
		G to A	GCG to GCA	sSNP	8946	82%	na
		T to C	TAT to TAC	sSNP	8982	83%	na
		A to G	ACA to ACG	sSNP	9006	82%	na
		G to A	AGC to AAG	nsSNP	9011	83%	S3004N
		C to T	GTC to GTT	sSNP	9024	85%	na
		C to G	GTC to GTG	sSNP	9042	86%	na

Abbreviation: na, not applicable.

^a^ sSNP stands for synonymous single nucleotide point mutation and nsSNP stands for non‐synonymous single nucleotide point mutation.

^b^ Position of mutation(s) is based on genome sequence of parental *B. subtilis* GM_03.

## 4 Discussion

Plastic degradation by environmental microbes could represent an untapped resource for potential novel solutions to the mounting plastic waste crisis worldwide. In this study, we demonstrated for the first time that a microbe isolated from the *G. mellonella* larvae microbiome ( *B. subtilis* GM_03) was capable of degrading two different plastics (LDPE and PU). Plastic degradation was demonstrated across LDPE and PU films, powders and suspensions, using a variety of different methods including visualisation of the clearance zone, SEM, bacterial survival assays and tension testing. Genomic analysis of *B. subtilis* GM_03 revealed putative genes potentially responsible for PU and LDPE degradation, including an oxidoreductase (AOALCJB_01849), an esterase (AOALCJB_01862) and a deacetylase (AOALCJB_03215). The oxidoreductase gene AOALCJB_01849 showed 100% query coverage and 87.67% similarity to Oxr‐1, an oxidoreductase from *Bacillus velezensis* previously shown to facilitate Impranil degradation (Gui et al. 2023). Additionally, an esterase gene in *B. subtilis* GM_03 shares homology with an esterase from *Delftia acidovorans* , which has been shown to degrade solid PU (Nomura et al. 1998). Similarly, the deacetylase gene in *B. subtilis* GM_03 exhibited 31% similarity to a deacetylase from the fungal strain *Rhizopus oryzae* , which is involved in LDPE degradation (Seenivasagan et al. 2022). Despite being a cross‐kingdom match, this level of conservation suggests that the deacetylase in *B. subtilis* GM_03 may play a key role in LDPE degradation, warranting further investigation. Overall, this unique suite of genes may drive this organism's rare ability to degrade more than one plastic.

Using directed evolution, we have successfully demonstrated that the PU plastic degrading capacity of *B. subtilis* GM_03 can be greatly improved. Although the directed evolution experiment was run for a relatively short number of passages (*n* = 4), the time scale of 13 days prior to passage one would result in around 100 generations (Maughan and Nicholson 2011) and was sufficient to see an improvement in degradation rate and induce adaptive mutations. This evolutionary timeframe compares well with previous directed evolution studies, where *B. subtilis* was grown 45 generations and compensatory genomic mutations could be identified (Ding et al. 2024).

Comparative genomic analysis of the evolved population (Passage 4) revealed mutations in genes encoding long‐chain fatty acid CoA ligase (*fadD*), surfactin synthetase subunit 2 (*srfAB*), two‐component sensory histidine kinase (*citS*), and oligopeptide binding protein (*appA*). The involvement of *fadD* and the long‐chain fatty acid metabolism in efficient plastic degradation is perhaps unsurprising, as this has long been considered one of the major ways plastics can enter the bacterial central metabolism. For example, carboxylated n‐alkanes resemble fatty acids, so they can easily enter the β‐oxidation pathway in bacterial cells (Yoon et al. 2012; Eubeler et al. 2010; Gewert et al. 2015). Surfactin is a secreted protein that reduces water tension, enhances salt tolerance and exhibits antibacterial properties. The *srfAB* gene is part of the *srfAA‐AD* operon responsible for synthesising surfactin (Qiao et al. 2024). Detection of 11 independent mutations in the *srfAB* gene in the Impranil evolved population indicates that they are being heavily selected for and that surfactin production plays an important role in PU degradation. Previously, it has been shown that the link between surface tension and plastic degradation; the addition of bio‐surfactin obtained from *B. licheniformis* enhanced LDPE degradation (Mukherjee et al. 2016). The mutation detected in the *citS* gene may result in a disruption of the cell's ability to take up citrate, forcing it to increase utilisation of Impranil or, alternatively, because of its location adjacent to a carbon catabolite repression protein A (CcpA) binding site, it may have enhanced citrate uptake, thereby accelerating initial growth (Repizo et al. 2006). Finally, the mutation in the *appA* gene rendering the membrane‐bound binding protein AppA and its associated oligopeptide permease systems in *B. subtilis* inactive indicates its ability to import short peptides is altered.

## 5 Conclusions

In summary, this study identified a *B. subtilis* strain from *G. mellonella* larvae microbiome that is capable of degrading two types of plastics (LDPE and PU) and highlights an underexplored avenue to solving mixed waste plastic recycling issues. *B. subtilis* has previously been isolated from many environments, including both terrestrial and aquatic environments (Earl et al. 2008) and has been associated with both LDPE (Yao et al. 2022) and PU degradation separately (Rowe and Howard 2002). Although *Bacillus* sp. has been isolated from the gut of *G. mellonella* larvae after feeding with PE (Cassone et al. 2020; Lou et al. 2020), this study is the first to demonstrate that *B. subtilis* from *G. mellonella* is capable of degrading two types of plastics. Enzymes potentially involved in the degradation of both plastics were identified, and the relationship between long‐chain fatty acid utilisation, surfactin production, and enhanced PU degradation were highlighted and require deeper exploration. Further investigation into the metabolic processes underlying the dual capabilities of *B. subtilis* GM_03 is warranted, as the same enzymes and regulatory proteins may be implicated in both degradation pathways. Differential transcriptomics experiments could be performed to assess changes in gene expression during different plastic exposures to facilitate the further defined involvement of degradation pathways, for both the wild type strains and evolved mutants.

Our study has demonstrated evidence of microbial degradation of LDPE and PU including clearance zone visualisation, SEM and tensile testing. Although there are additional, more quantitative methods that have previously been used to demonstrate microbial plastic degradation, such as monitoring reduction of plastic weight (Hou et al. 2022; Abraham et al. 2017), these were not employed in this study, as weight loss can be confounded particularly with regards to microbial growth in biofilms (Howard et al. 2025) and also when using powders, which can be difficult to fully recover from solution, resulting in overestimation of weight loss (Tribedi and Sil 2013; Montazer et al. 2020; Obrador‐Viel et al. 2024). Although detailed chemical analysis of breakdown products was excluded from the scope of this study due to a focus on microbial screening, the breakdown products resulting from LDPE degradation by *G. mellonella* or microbes have previously been defined using gas chromatography mass spectroscopy (GC–MS) and high performance liquid chromatography (HPLC) and reported to include ethylene glycol, alkanes, esters, aldehydes and carboxylic acids (Cassone et al. 2020; Rong et al. 2024; Yun et al. 2023). Similarly, breakdown products of Impranil have previously been shown to include 1,6‐hexanediol, neopentyl glycol (Fuentes‐Jaime et al. 2022) adipic acid, 1,4‐butanediol and methylenedianiline derivatives (Ji et al. 2024).

Overall, this research underscores the importance of evaluating microbes for their ability to degrade multiple plastics, rather than focusing on a single type, as is commonly practised. Further exploration of multi‐talented microbes such as GM_03 could include assessing the microbial ability to degrade real‐world mixed plastics, such as those in mixed waste streams and multi‐layered plastics commonly used in packaging materials. Lastly, synthetic biology approaches could be employed to further engineer *B. subtilis* GM_03 for improved plastic‐degrading functionality under precision fermentation conditions and for large‐scale industrial fermentation tanks. For this, the optimisation of the growth conditions required for peak plastic degradation conditions, including pH, media composition and temperature (Tirkey and Upadhyay 2025) would also be beneficial.

## Author Contributions

**Amy K. Cain**, **Ram Maharjan**, **Hue Dinh**, **Fiona S. B. Facey:** conceptualisation. **Fiona S. B. Facey**, **Ram Maharjan**, **Aidan P. Tay**, **Amy K. Cain**, **Hue Dinh**, **Ian T. Paulsen**, **Jason S. Buchanan**, **Luke A. Connal:** investigation. **Fiona S. B. Facey**, **Hue Dinh**, **Ram Maharjan**, **Amy K. Cain:** writing. All authors: reviewing and editing.

## Conflicts of Interest

The authors declare no conflicts of interest.

## Supporting information


**Data S1:** emi470216‐sup‐0001‐supinfo.docx.

## Data Availability

Raw sequencing data are available through the European Nucleotide Archive (ENA) Project PRJEB85982, https://www.ebi.ac.uk/ena/browser/view/PRJEB85982.
