# Supplementary material for: Characterising the Multiple‐Plastic Degrading Strain of Bacillus subtilis GM_03 From the Galleria mellonella Microbiome
Source: Environ Microbiol Rep. 2025 Oct 20;17(5):e70216. doi: 10.1111/1758-2229.70216 (PMC12537272; doi:10.1111/1758-2229.70216)
Supplement: Supplementary file 1 — Data S1: emi470216‐sup‐0001‐supinfo.docx. [file EMI4-17-e70216-s001.docx]

# **Table S1** Output of 16s rRNA sequencing on six isolates from *G. mellonella* larvae, showing the genus identified, Query cover and % identity from BLASTn and the sequence GenBank accession number**.**

| **Sample identifier** | **Identified genus** | **Query cover**  **%** | **Percent identity from BLASTn**  **%** | **GenBank accession number** |
| --- | --- | --- | --- | --- |
| 1 | *Dermacoccus sp.* | 98 | 97.86 | PV224562 |
| *2* | *Bacillus sp.* | 98 | 99.26 | PV224564 |
| *3* | *Bacillus sp.* | 99 | 99.33 | PV224566 |
| *^4^* | *Dermacoccus sp.* | 99 | 97.59 | PV224569 |
| *^5^* | *Enterococcus sp.* | 98 | 89.26 | PV224570 |
| *^6^* | *Micrococcus sp.* | 99 | 99.83 | PV224571 |


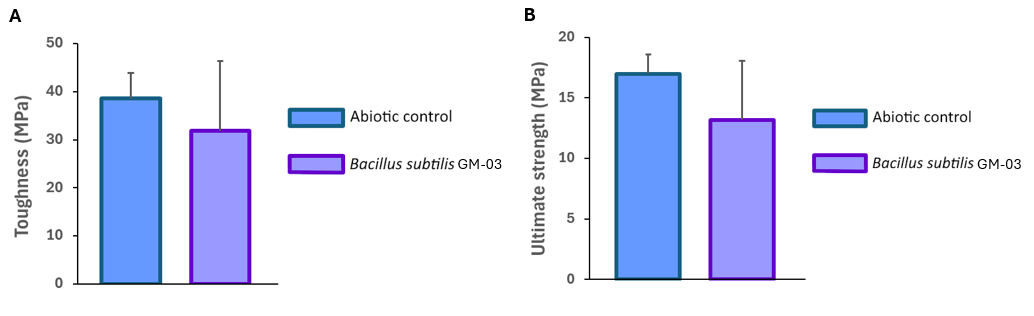
 **Fig. S1**. With PE as the only carbon source when incubated with *B. subtilis* for 95 days, tensile testing results with five technical replicates show in **A** a 17.56% reduction in average toughness from 38.56 MPa to 31.79 MPa, and **B** a 22.38% reduction in average ultimate strength from 16.99 MPa to 13.19 MPa

**Table S2.** Details of similarity of genes in *B. subtilis* GM_03 compared with published PU and PE degrading genes from PlasticDB ^1^


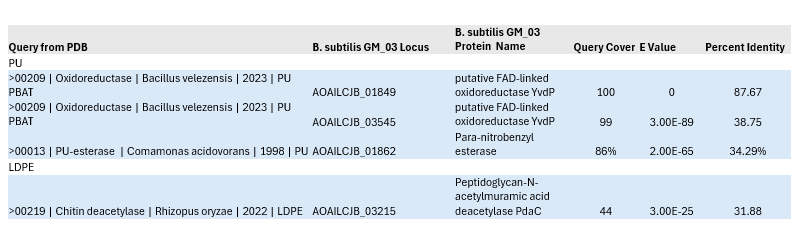


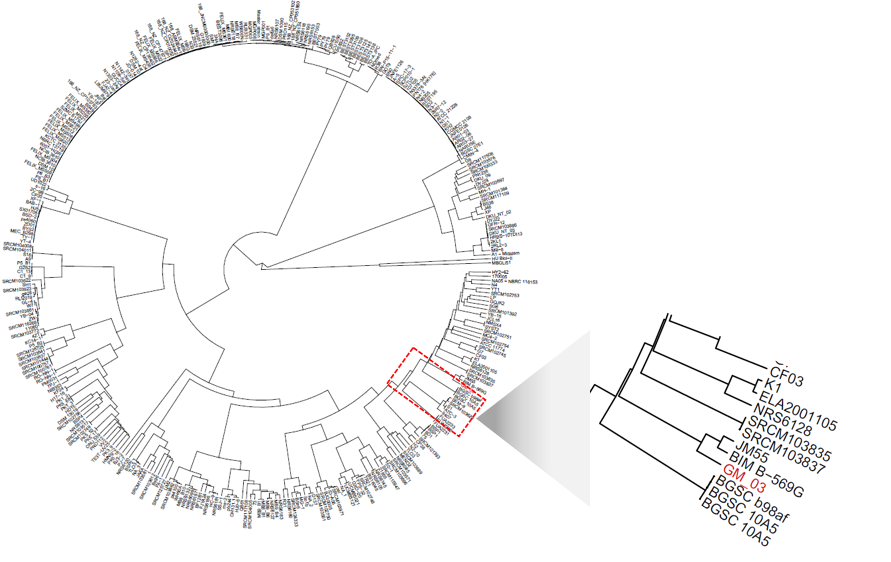


**Fig S2**. Phylogenetic Tree of 343 strains of *B. subtilis* available in NCBI. The dashed box and zoomed inset indicate the closest strains to the *G. mellonella* isolate *B. subtilus* GM-03 .


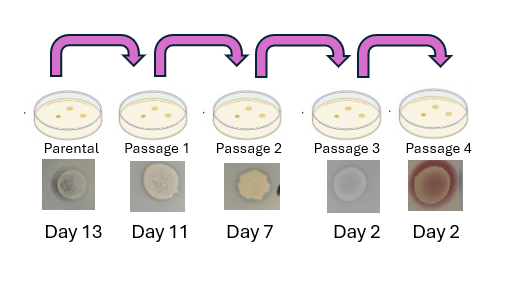


**Fig S3.** Workflow of directed evolution of *B. subtilis* GM_03 isolate from *G. mellonella* on agar plates containing Impranil and sodium citrate. Showing clearance zone and days to initial clearance from parental strain through to passage 4 Clearance zone in directed evolution experiment showing improvement in clearance time across passages from parental strain through to passage 4.

1 Gambarini, V. *et al.* PlasticDB: a Database of Microorganisms and Proteins Linked to Plastic Biodegradation. *Database* **2022** (2022). [https://doi.org:10.1093/database/baac008](rewritten://9f3719bf-6be1-460e-8260-d39d28a03f22)
